# Inhibition of Murine Systemic Leishmaniasis by Acetyl Salicylic Acid via Nitric Oxide Immunomodulation

**Published:** 2012

**Authors:** H Nahrevanian, M Jalalian, M Farahmand, M Assmar, AR Esmaeili Rastaghi, M Sayyah

**Affiliations:** 1 Department of Parasitology, Pasteur Institute of Iran, Tehran, Iran; 2 Department of Microbiology, Islamic Azad University of Lahijan, Gilan, Iran; 3 Department of Physiology & Pharmacology, Pasteur Institute of Iran, Tehran, Iran

**Keywords:** ASA, Balb/c, *Leishmania major*, NO, Immunotherapy

## Abstract

**Background:**

The purpose of this study was to evaluate antileishmanial effects of ASA via NO pathway in *Leishmania major* infected Balb/c mice. Moreover, toxicity and pathological consequences of ASA administration were investigated.

**Methods:**

Balb/c mice were infected with *L. major* and ASA was inoculated orally after lesion appearance for its ability to modulate NO and to modify *Leishmania* infection in host, in order to evaluate the effects of NO production on size and lesion macroscopy, delay of lesion formation and proliferation of amastigotes inside macrophages. Liver, spleen, and lymph nodes were also studied as target organs to detect amastigotes. In addition, plasma was investigated for NO induction using Griess microassay.

**Results:**

ASA increased NO production in plasma of both naïve and *Leishmania* test groups at the ultimate of the experimental period. A decline was observed in proliferation of amastigotes inside macrophages of test group when compared with control one. ASA reduced lesion size, inhibited *Leishmania* visceralisation in spleen, lymph node, and decreased hepato/splenomegaly in ASA treated animals.

**Conclusions:**

Some antileishmanial effects of ASA by NO-modulation were indicated during systemic leishmaniasis in mice. Despite slight effects on lesion size, ASA decreased parasite visceralization in target organs and declined their proliferation inside macrophages. Therefore, ASA may be indicated to inhibit systemic leishmaniasis via NO pathway in mice model.

## Introduction

Leishmaniasis is a zoonotic disease caused by *Leishmania* (*L*.) parasites, ranging from self-healing cutaneous lesion to severe and non-healing disseminated cutaneous (CL) or visceral leishmaniasis (VL). Cutaneous leishmaniasis is a chronic infectious and granulomatous disease caused by *Leishmania* parasites. Parasite can multiply in macrophages (MQs) and the clinical spectrum of the disease is due the severity of the immune response of the host (1).

Immune responses during leishmaniasis include antibodies, cytokines, immune cell, mediators, and acute phase proteins. *L. major* infection in various inbred strains of mice has been used extensively to study the immunological events that control the *in vivo* development of Th1 versus Th2 type responses. Activated MQs participate in the inflammatory response by releasing chemokines and factors that recruit additional cells to site of infection (2). There are several experimental evidences that nitric oxide (NO) is involved in the microbiocidal activity of MQs against a number of intracellular pathogens including *L. major, Trpanozoma cruzi* and *Toxoplasma gondii* (3). NO is an inorganic free radical which is remarkably versatile biological messenger. NO is a short- lived biological mediator produced by many cell types to induce many functions and it acts as both pro- and anti-inflammatory agents. The mechanisms that underline these effects remain poorly defined. Some chemical properties of NO as a cytotoxic and antipathogenic agent released during an inflammatory response (4). In addition to NO, some microelements are *major* acute phase parameters present in normal serum, which increases significantly after most forms of infections as a non-specific innate defence mechanism of the host. The data have been revealed a correlation of NO in some infections. This may clarify the involvement of NO as a *major* immune element during infection; however, it is not justified, whether the NO production is beneficial or detrimental to the host (5).

Acetyl salicylic acid (ASA) can inhibit inflammatory reactions and platelet aggregation, but little is known about its efficacy in treatment of leishmaniasis (6). ASA is unique among the nonsteroidal anti-inflammatory drugs (NSAIDs); thus, they have popularized the notion of inhibiting prostaglandin (PG) biosynthesis as a common anti-inflammatory strategy (7). Recently, NO-donating NSAIDs are emerging as an important novel pharmacological class that has already entered the phase of clinical testing (8). Hybrid drug NO-ASA continues to attract intense research from chemists and biologists alike. It consists of ASA and a -ONO2 group connected through a spacer and is in preclinical development, however, there are some contrary reports to current beliefs (9). ASA is known to exert antioxidant effects by unidentified mechanisms (10). The data from recent experiments proposing that one of ASA roles in inflammation is the induction of NO, which potently inhibits leukocyte /endothelium interaction during acute inflammation. It will be argued that this NO-inducing effect is exclusive to ASA unique ability, among the traditional anti-inflammatory drugs (11).

In this study, a novel ASA mode of action will be discussed and its new pathway as antileishmanial agent will be investigated during *Leishmania* infection.

## Materials and Methods

### Animals

Male inbred Balb/c mice (supplied by Karaj Laboratory Animal Unit, Pasteur Institute of Iran) were used in this study. The initial body weight was 14±0.4 (mean ± standard error of mean, SEM) and mice were housed at room temperature (20-23 °C) on a 12 h light and 12 h dark cycle, with unlimited access to food and tap water. Experiments with animals were done according to the ethical standards formulated in the declaration of Helsinki, and measures taken to protect animals from pain or discomfort. It has been approved by institutional Ethical Review Board, in which the work was done.

### In vitro cultivation of L. major

The *L. major* used in this study was Iranian strain MRHO/75/ER was obtained from Department of Parasitology, Pasteur Institute of Iran. The parasites were maintained by regular passage in susceptible Balb/c mice. The parasites were cultured in the RPMI 1640 medium supplemented with 10% fetal bovine serum (FBS), 292 g/ml L–glutamine and 4.5 mg/ml glucose. Under these culture conditions, the stationary phase of parasite growth was obtained in 6 days as determined (12).

### Infection of Balb/c mice with L. major

Promastigotes of *L. major* were harvested from culture media, counted, and used to infect Balb/c mice. The base of the tail was injected intradermally with inoculums of 2×10^6^ promastigotes. The animal experiments were performed once in 4 groups (n = 10 mice/group) considering time, budget and long-period monitoring of animals according to the ethical issues for sample size and replication. The *Leishmania* infection was carried out in experimental animals and terminated at week 13 after injection.

### Determination type of inoculation process

ASA (Sigma) was dissolved in 40% ethanol to make a concentration of 100 mg/ml and applied in two routes as oral and injection groups. Toxicity assays were used to determine possible detrimental side effects. The optimum oral route of inoculation was applied in following experiments.

### Dose determination of ASA

ASA was tested in three doses as low (100), medium (200) and high (400 mg/Kg of body weight) in naïve animals. According to toxicity assays, which presented no negative side effects, conclusively the high dose was selected for further studies.

### Anti-leishmanial assay of ASA

ASA (400 mg/Kg of body weight) was inoculated orally into mice after lesion appearance using gavages (once a day up to 13 weeks). The animals used in this experiment were divided into 4 groups including Group 1 (control naïve), Group 2 (test naïve), Group 3 (control *L. major*) and Group 4 (test *L. major*).

## Assessment of Pathology

### Measurement of lesion size

Lesion size was measured at every other week after inoculation in millimeters (mm) by a digital caliper (Chuan Brand, China) in two diameters (D + d) at right angles to each other, and the size was determined according to the formula: (D + d)/2 (13).

### Microscopical examination of smear

The clinical diagnosis was confirmed by laboratory demonstration of the parasite in the lesion by marking stained smear at end of the experimental period. Lesions were cleaned with ethanol and punctured at the margins with a sterile lancet and exudation material was smeared.

### Impression smear preparation

Impression smear were prepared from liver, spleen and lymph nodes by placing a small piece of tissue between two glass slides and pushing them in different directions. The smears were air dried, fixed by methanol, and stained with Giemsa for detection of amastigotes by light microscopy (14, 15).

### Measurement of amastigote's proliferation

The proliferation of parasite was evaluated by counting of amastigotes inside MQs on Geimsa stained lesion smear at the end of the experimental period. Five random MQs were selected; counted and mean percentages were calculated as indicators for the degree of proliferation in amastigotes inside each MQ (15).

### Assessment of degree of hepato/splenomegaly

Entire livers and spleens were removed post-mortem at the end of the experimental period from mice after induction of terminal anesthesia by inhalation of diethyl ether (Sigma). Organ wet weights were measured and compared with controls as indices for degree of hepato / splenomegaly.

### Measurement of survival rate and body weight

Survival rate was presented as the percentage of surviving experimental mice at every other week after inoculation; the significance of differences was determined by ANOVA test and compared with concurrent appropriate vehicle-treated *Leishmania* and control groups. Body weight was measured initially and at different time of experiment using a top pan balance (OHAUS Scale Corp., USA).

### NO detection by Griess micro assay

The Griess reaction was adapted to assay nitrate as described previously. Nitrate was determined indirectly by the Griess micro assay (GMA), as the nitrite produced from nitrate when incubated with nitrate reductase. Standard curves (range 1-60 nmol/ml) for sodium nitrite (NaNO_2_, Sigma) and sodium nitrate (NaNO_3_, Sigma) were prepared in both plasma and supernatant fluid from tissue homogenates, using pooled surpluses from uninfected mouse samples. Sixty µl samples were treated with 10 µl nitrate reductase (NAD[*P*]H *Aspergillus* species 5U/ml, Sigma) and 30 µl NADPH β-nicotinamide adenine dinucleotide phosphate (1.25 mg/ml, Sigma Diagnostics, St. Louis, USA). Tow hundred µl Griess reagent (5% phosphoric acid, 1% sulfanilic acid and 0.1% N (1-naphthyl-1)-ethylendiamine dihydrochloride (NED), all from Sigma, dissolved in 100 ml deionised water) was then added and proteins subsequently precipitated by 200 µl trichloroacetic acid 10%, (BDH, England). Tube contents were vortex mixed then centrifuged at 13,400 RCF (Microcentrifuge, Sigma, UK). Duplicate 200 µl samples of supernatants were transferred to a 96-well flat-bottomed microplate (Costar, USA) and absorbances read at 520 nm using a microplate reader (BioTek, USA). Values for the concentration of nitrite assayed were calculated from standard calibration plots for NaNO_2_ and NaNO_3_ following nitrate reductase action (16).

### Statistical analysis

Values are presented as the mean ± SEM for groups of *n* samples. The significance of differences was determined by Analysis of Variances (ANOVA) and Student's *t*-test using Graph Pad Prism Software (Graph Pad, San Diego, California, USA) and Microsoft Office Excel 2007.

## Results

**Fig. 1:**
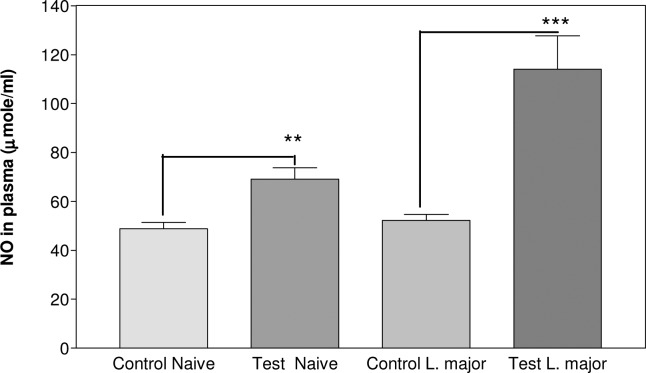
NO Production in plasma of experimental groups of Balb/c mice. NO Production was measured by GMA in plasma of entire groups at the end of the experimental period. Significant analysis (٭٭*P*<0.01, ٭٭٭*P*<0.001) was determined by Student's *t-test* using Graph Pad Prism (n=10 mice/group)

NO production increased by ASA in plasma of both naïve (69.1±4.7, *P*<0.01) and *Leishmania* test (114.1±13.6, *P*<0.001) groups at the end of the experimental period (Fig. 1).

**Fig. 2:**
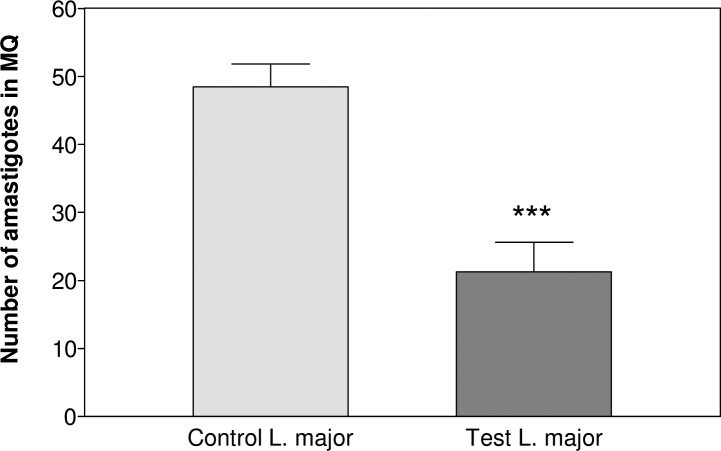
Comparative proliferation of amastigotes inside MQs of CL lesions from *L. major* infected mice. The proliferation of parasite was evaluated by counting amastigotes inside random MQs on Geimsa stained smears of CL lesions in *Leishmania* group at the end of the experimental period. Significant analysis (٭٭٭*P*<0.001) was determined by Student's *t-*test using Graph Pad Prism (n=10 mice/group)

**Fig. 3:**
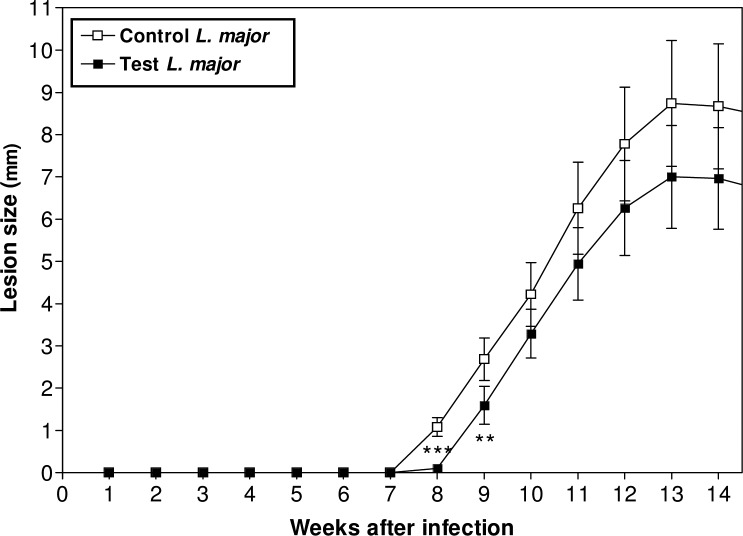
ASA effects on lesion size and comparison with control of *Leishmania* group Significant difference (٭٭*P*<0.01, ٭٭٭*P*<0.001) was determined by Student's *t-*test using Graph Pad Prism (n=10 mice/group)

Comparative study on CL lesions of infected Balb/c mice with *L. major* represented a sharp decline (*P*<0.001) in proliferation number of amastigotes inside MQs (Control 48.5±3.3, Test 21.3±4.3) (Fig. 2). ASA reduced lesion size in test group with a significant difference (*P*<0.001) after 7 weeks of *Leishmania* inoculation (Control 8.7±1.5, Test 6.9±1.2) (Fig. 3).

**Fig. 4:**
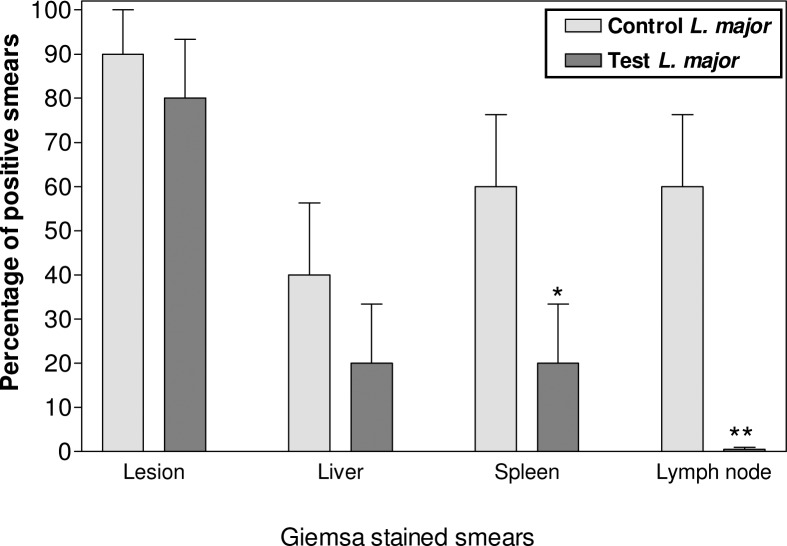
Amastigotes in smears of target tissues in mice infected with *L. major* The percentage of positive smears was compared between control and test groups of lesion, and target tissues. Significant differences (٭*P*<0.05, ٭٭*P*<0.01) was determined by Student's *t-*test using Graph Pad Prism (n=10 mice/group) As a side effect of ASA application, a reduction up to 30% in survival rates was found in naïve and *Leishmania* test groups after 11 weeks of infection and a slight weight loss (*P*<0.05) was observed in both naïve and *Leishmania* test groups after 6 weeks of inoculation.

**Fig. 5:**
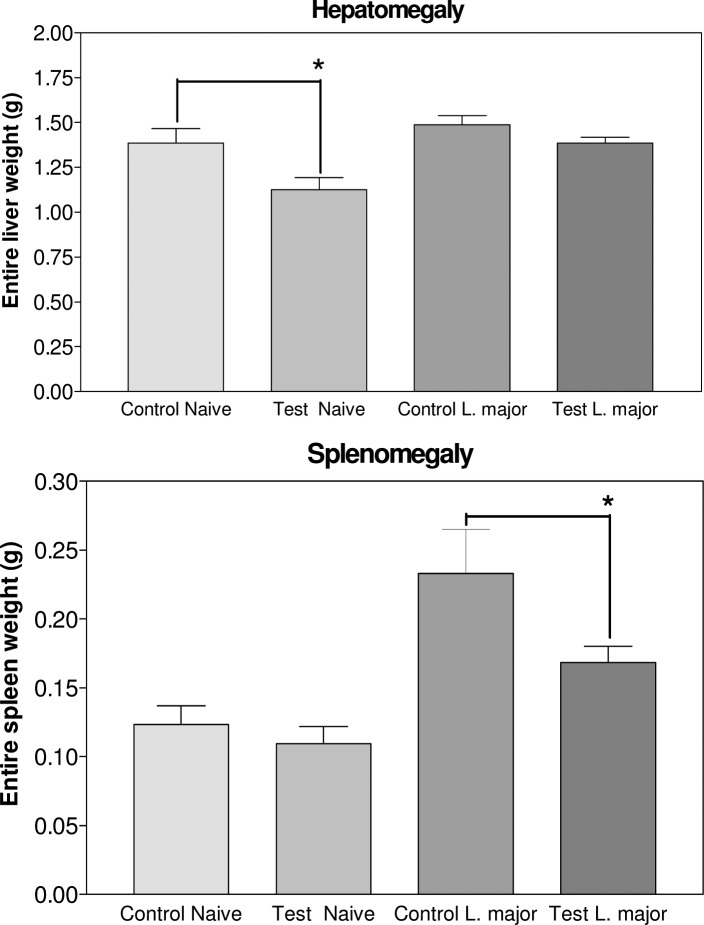
ASA effects on hepatomegaly and splenomegaly in naïve and *Leishmania* groups Significant variation (٭*P*<0.05) was detected by Student's *t-*test using Graph Pad Prism (n=10 mice/group)

ASA inhibited *Leishmania* visceralisation which was indicated by observation of amastigotes in spleen (Control 60.0±16.3, Test 20.0±13.3; *P*<0.05) and lymph node (Control 60.0±16.3, Test 0.5±0.05; *P*<0.01) smears (Fig. 4). In addition, ASA reduced hepatomegaly in naïve group (Control 1.4±0.08, Test 1.1±0.06; *P*<0.05), inhibited hepatomegaly and decreased splenomegaly (Control 0.23±0.03, Test 0.16±0.01; *P*<0.05), because of leishmaniasis in *Leishmania* group (Fig. 5).

## Discussion

This experiment was a part of a continuous study on the role of NO against intracellular parasite *Leishmania* (5, 17–18). In previous study, data revealed an association between increases in NO level with the pathology of disease in Balb/c mice infected with *L. major*.

ASA induced significantly plasma NO in both naïve and *Leishmania* groups, which may reduce visceral proliferation of parasites in the *Leishmania* infected hosts. Assessment of ASA represented that this medication reduced (almost 50%) the proliferation of amastigotes inside MQs, indicating its positive effects on CL in long-term infection. No significant reduction in body weight indicated its low pathophysiological side effects in test groups. In addition, the survival rate in mice did not show significant changes in infected group, however a minor decrease has been found in the infected test group. Moreover, ASA reduced lesion size of *Leishmania* in mid-infection until final stage of disease; it clearly presented its positive effects on parasite proliferation in smears of lesion, liver, spleen, and lymph node. The degree of hepato / splenomegaly was also decreased by ASA, indicating its possible antipathological characteristics.

In addition, survival of *Leishmania* parasite inside the MQ and its proliferation was affected by NO and an inconsistent relationship was evident between the NO modulation and pathological changes in host (15). A partial role for NO is highlighted here, which is in accordance with published reports describing that intracellular control of leishmaniasis in the human and murine models is partly NO dependent, and has been confirmed by several laboratories (19–21). Therefore, some studies are in agreement with current investigation. *Leishmania major* infected C57BL/6 NOS deficient mice developed more severe skin lesions with strikingly higher numbers of parasites. It was concluded that during CL, NO counteracts the recruitment of granulocytes, and thereby limits the severity of the skin lesions (22). Some data suggested NO facilitates the parasite killing by MQs via monocyte chemo attractant protein-1 (MCP-1) -mediated stimulation (23). Immune control of *Leishmania* growth absolutely requires expression of inducible NO synthase (iNOS) and subsequent production of NO during chronic *L. major* infection of C57BL/6 resistant mice (24).

Moreover, ASA presented its ability to elevate NO concentration in plasma during systemic leishmaniasis in mice. The results of this study were supported by de Souza et al. (25), which presented NO-production by infected macrophages were correlated with resistance in the human and murine models of CL. In a first test was performed in humans to indicate whether ASA increases NO formation; nonetheless, these data contribute to the hypothesis that ASA has additional beneficial effects mediated through NO formation (26).

In contrary, some reports do not support this hypothesis. In a study determined the clinical responses of NO plus cryotherapy, results did not show any more effectiveness of combination therapy consisting 3% NO cream and cryotherapy for the treatment in patients with CL (27). Although *Leishmania* amastigotes were able to reduce NO production in host, the interference with this cytotoxic mechanism was not sufficient to permit the survival of *L. mexicana* (28).

It seems application of ASA could decrease parasite visceralization in target organs as well as declining its proliferation inside MQs with less effects on lesion size. In several reports, there is a weak indication for NO efficacy by local NO inducer during leishmaniasis *in vivo* on lesion during CL. In addition, some negative aspects were also observed by ASA application on hepato / splenomegaly, survival rate and body weight.

This study reveals other concept of the elusive properties of ASA to affect *Leishmania* through immune modulation, suggesting that ASA has some other modes of action depending on the disease state. It is indicated ASA may be applied for inhibition of systemic leishmaniasis *via* NO pathway in Balb/c mice infected with *L. major*, however more studies are required to clarify this concept on different *Leishmania* parasite species and several susceptible and resistant hosts.
